# Spatiotemporal Variation Analysis of the Fine-Scale Heat Wave Risk along the Jakarta-Bandung High-Speed Railway in Indonesia

**DOI:** 10.3390/ijerph182212153

**Published:** 2021-11-19

**Authors:** Xin Dai, Qingsheng Liu, Chong Huang, He Li

**Affiliations:** State Key Laboratory of Resources and Environmental Information System, Institute of Geographic Sciences and Natural Resources Research, Chinese Academy of Sciences, Beijing 100101, China; daixin1129@163.com (X.D.); lih@lreis.ac.cn (H.L.)

**Keywords:** heat waves, hazard, exposure, vulnerability, remote sensing, Jakarta-Bandung high-speed railway

## Abstract

As a highly important meteorological hazard, heat waves notably impact human health and socioeconomics, and accurate heat wave risk identification and assessment are effective ways to address this issue. The current spatial scale of heat wave risk assessment is relatively coarse, hardly meeting fine-scale heat wave risk assessment requirements. Therefore, based on multi-source fine-scale remote sensing data and socioeconomic data, this paper evaluates the heat wave risk along the Jakarta-Bandung high-speed railway, obtains the spatial distribution of heat wave risk in 2005, 2014 and 2019, and analyzes spatiotemporal risk variations over the past 15 years. The results show that most high-risk areas were affected by high-temperature hazards. Over time, the hazard, exposure, vulnerability and risk levels increased by 25.82%, 3.31%, 14.82% and 6.97%, respectively, from 2005–2019. Spatially, the higher risk in the northwest is mainly distributed in Jakarta. Additionally, a comparative analysis was conducted on the risk results, and the results showed that the 100-m scale showed more spatial differences than the kilometer scale. The research results in this paper can provide scientific advice on heat wave risk prevention considering the Jakarta-Bandung high-speed railway construction and regional economic and social development.

## 1. Introduction

In the Intergovernmental Panel on Climate Change (IPCC) Special Report on Global Warming of 1.5 °C, it is estimated that human activities have caused approximately 1.0 °C of global warming over preindustrial levels, with a likely range from 0.8 °C to 1.2 °C [1]. Global warming is projected to intensify heat wave events, with a trend toward an increased frequency, intensity and duration [2,3,4]. A total of 70,000 people perished in a heat wave in Europe in 2003 [5], and a heat wave lasting three weeks in Russia caused approximately 56,000 deaths in 2010 [6], while a heat wave in Pakistan killed more than 200 people within a week in 2015 [7]. In April 2016, heat waves occurred in several countries in Southeast Asia, severely affecting crop growth and causing social hardship [8]. The direct adverse effects of heat waves also include: power shortages [9,10], agricultural losses [11,12], and infrastructure damage [13]. The impact of heat waves on human health and global socioeconomic activities is becoming increasingly severe and is now the leading cause of weather-related illnesses and death globally [5,14]. Therefore, heat waves are highly valued in the field of extreme weather, and the identification and assessment of the heat wave risk (HWR) have become the basis and important foundation for disaster mitigation and development of emergency management responses.

The Jakarta-Bandung high-speed railway (JBHSR) is Indonesia’s first high-speed railway, connecting Jakarta with Bandung, and is one of the key nodes along the Belt and Road initiated by China, with an important demonstration and leading role [15]. The JBHSR is located close to the equator, and the region remains hot year round. Regional disasters are frequent, with meteorological and hydrological disasters accounting for 77% of all disasters in Indonesia between 1815 and 2011 [16], and among these disasters, heat waves represent one of the most frequent meteorological disasters. At present, there are few reports on heat wave studies in the area, with a notable lack of fine-scale HWR assessment results to scientifically guide local governments, people and construction companies in the development of effective HWR prevention and response measures. Moreover, high-speed railway construction certainly produces great demographic, economic and social development along the route, which can cause spatiotemporal HWR changes. Therefore, it is important to conduct HWR assessment in this region.

A review of the current literature on HWR assessment worldwide reveals the following problems: (1) In terms of the time scale, most of the studies on HWR changes at long time scales are based on meteorological station data, and only indicators of the heat wave intensity, frequency and duration are chosen [17,18,19,20,21], or indicators such as the effective cumulative temperature calculated considering meteorological station temperature data [22] to study heat waves in a particular region over decades. However, studies based on meteorological station data consider only one station to represent the temperature of the whole city, or when the study area is large, the variability in heat waves is mostly studied via interpolation of station-measured temperatures [20,21,23]. Although the above methods can suitably analyze changes over time on a yearly scale, the spatial scale is coarse (10–50 km), and only analyses from the perspective of heat wave hazards have been conducted. This ignores the uneven distribution of the temperature within cities caused by the topography, natural environment and urban heat island effect and fails to reveal the spatial differences in temperature within regions. (2) In recent years, the accelerating urbanization process has led to further intensification of the urban heat island effect. Kazak utilized land use data to construct a decision support system and found that areas with a concentration of large cities have higher levels of potential UHI exposure [24]. Numerous scholars have shown that urban heat islands have a synergistic relationship with heat waves [25,26,27]. During heat waves, urban residents are exposed to more severe and sustained heat stress [28], and the morbidity and mortality of residents are increased [29,30,31]. Fischer considered urban models and found that heat stress in cities would be greatly increased during heat waves [32]. Thus, humans, as the main bearers of heat waves are also increasingly affected by heat waves. In recent years, an increasing number of scholars have paid attention to the impact of heat waves on human health [33,34,35]. Studies on the impact of heat waves on human health should consider not only the impact of heat on humans but should also include a comprehensive assessment of the HWR in conjunction with factors such as natural and socioeconomic environments [5,36,37]. Estoque considered the land surface temperature (LST), population and economy factors [33]. Jackson selected sociodemographic, ambient temperature distribution, and shelter availability factors [36]. However, in this type of study, the risk of heat waves is usually studied for a single heat wave event or on a one-year time scale, and only the spatial distribution of high- and low-risk areas can be obtained. A multiyear (long time scale) HWR study provides a more realistic meaning, because it not only clarifies the spatial distribution of high- and low-risk areas but also identifies the overall HWR trends and the evolution of risk hotspots and predicts future risk trends. In conclusion, in regard to the spatiotemporal scale, the current research does not satisfy the realistic guidance needs of regional disaster prevention and mitigation.

The risk assessment along the JBHSR has not been studied until now. Only a few scholars have conducted some studies on the central cities (provincial capitals or national capitals) in Indonesia, covering a few themes, such as land use, urban heat island, and natural hazard vulnerability [38,39]. Relevant studies at the kilometer scale have been carried out for Indonesia regions in our previous work, which better represent the spatial differences in regional HWR and can be applied to regional disaster prevention and mitigation guidance. However, it is found that, spatially, the kilometer scale cannot reflect the spatial differences within a small region, and temporally, it cannot reflect the trend of risk changes and predict the future development trend of risk.

Therefore, this paper, after interviewing relevant experts many times, determines an index system for the HWR, applies Landsat data to obtain finer-scale LST data via inversion, and combines multisource fine-scale remote sensing data to carry out a study on the spatiotemporal changes in the HWR at the 100-m scale over the past 15 years (2005, 2014 and 2019). This study can provide a reference for regional disaster prevention and mitigation decisions, scientific warning and prevention systems of high-temperature risks and implementation of adaptation measures.

## 2. Materials and Methods

### 2.1. Study Area

**Figure 1:**
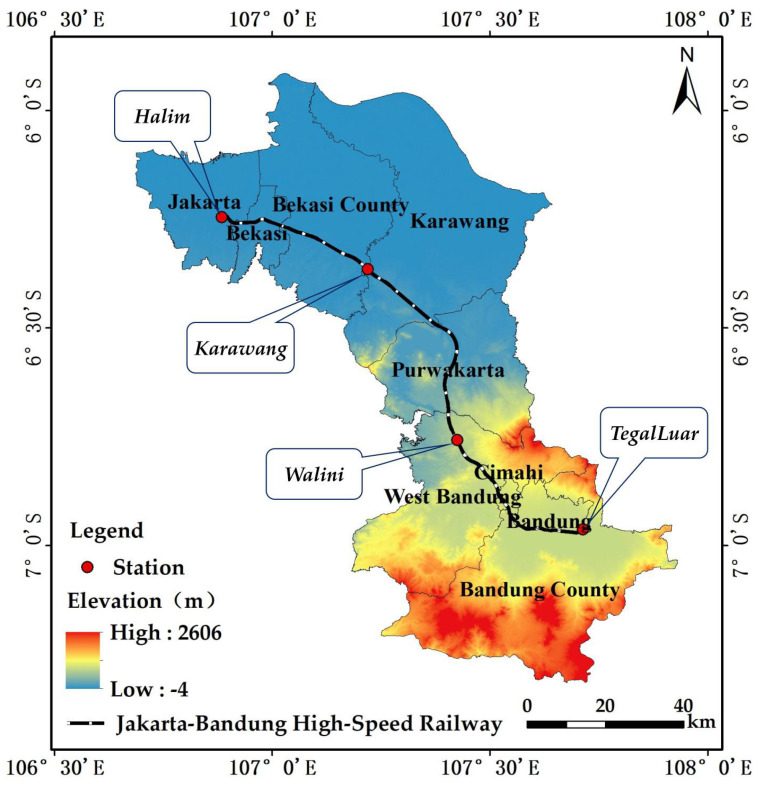
The location of the study area.

The JBHSR is located in northwestern Java, Indonesia, between 5°54′–7°19′ S and 106°42′-107°54′ E (Figure 1). The region is bordered by Banten Province in the west and the Java Sea in the north, and the JBHSR connects Jakarta, the capital of Indonesia, with Bandung, the capital of West Java, passing through Bekasi city, Bekasi Regency, Karawang Regency, Puwakarta Regency, West Bandung Regency, Cimahi city and Bandung Regency. The region is located in the tropics and is hot and humid year round. The highest temperature reaches 37.8 °C, the lowest temperature is 11.2 °C, and the relative humidity is above 70%. Due to the unique geographical location of the region, a rainy season, which is influenced by the northwest monsoon, lasting from November to March each year, and a dry season, which is influenced by the southeast monsoon, lasting from April to October each year, can be distinguished. The average annual precipitation exceeds 2000 mm. The regional landscape is complex, with alluvial plains, denuded remnant hills and gentle slopes, hills, low hills and intermountain basins, with the terrain gradually increasing from northwest to southeast [40].

**Figure A1:**
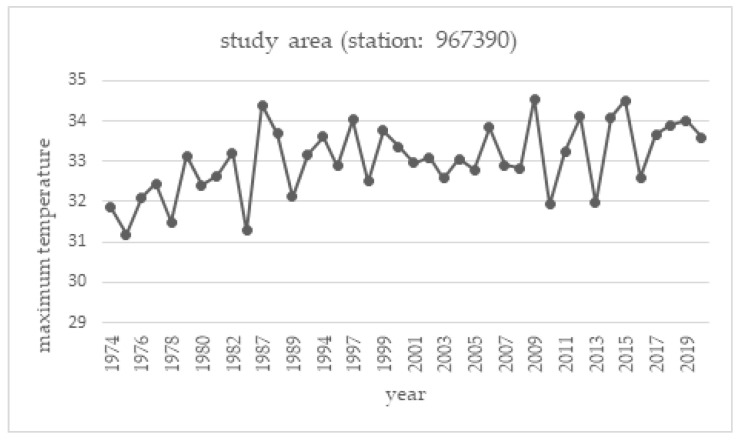
Average maximum temperature in September along the Jakarta-Bandung high-speed railway.

According to historical data acquired from meteorological stations in the study area, 51 heat waves occurred from 1973–2018 (station: Tangerang/Budiarto Airport; 967390), mainly concentrated from September-November, with 17 heat waves occurring in September, accounting for 33.3% of the total number of heat waves (Figure A1). After data cleaning and filtering of the remaining data, the historical average maximum temperature in September recorded at this site was obtained, indicating an upward overall trend.

### 2.2. Data Collection and Preprocessing

(1) Land surface temperature (LST) data. The LST was selected as a remote sensing indicator for HWR assessment [41]. Landsat-5 and Landsat-8 data were obtained to determine the LST via inversion with the radiative transfer equation method [42]. Statistical analysis revealed the highest frequency of heat waves in September in the study area, and only three years (i.e., 4 September 2005, 13 September 2014, and 11 September 2019) were selected for analysis, as Landsat data from 2010 or years near 2010 were more heavily influenced by clouds, and the data were of a poorer quality. The data with a spatial resolution of 30 m were resampled to 100 m.

(2) Population data. The data were freely downloaded from WorldPop [43] at a spatial resolution of 100 m [44]. This paper adopted population data for 2005, 2014 and 2019, including the population count, population of elderly individuals (>65 years) and population of young individuals (<5 years).

(3) Nighttime light (NTL) data. These data were derived from the corresponding dataset in the Figshare repository [45], which is a harmonized global NTL dataset from 1992–2018 [46]. The data resolution is 1 km, which was resampled to 100 m.

(4) Medical facility point data and road data. These data were sourced from OpenStreetMap [47] and extracted by the attribute in ArcGIS.

(5) The acquired remote sensing index data included normalized difference vegetation index (NDVI), modified normalized difference water index (MNDWI) and impervious surface data. NDVI and MNDWI data were obtained via the band math function in ENVI software (Boulder, CO, USA). Impervious surface data were first obtained with the band math function to retrieve NDBI data, and areas with NDBI values > 0 were then extracted as impervious surfaces, and areas with NDBI values < 0 were treated as pervious surfaces. The data with a spatial resolution of 30 m were resampled to 100 m.

### 2.3. Methods

#### 2.3.1. Heat Wave Risk Assessment Framework

Our study builds upon other previous related studies employing remote sensing and socioecological data [14,48]. The implemented heat wave HWR assessment approach relies on the IPCC conceptual risk framework reported in AR5, in which the risk is a function of hazard, exposure, and vulnerability [49].

(1) Hazard

A hazard refers to “the potential occurrence of a natural or human-induced physical event or trend or physical impact that may cause loss of life, injury, or other health impacts, as well as damage and loss to property, infrastructure, livelihoods, service provision, ecosystems, and environmental resources” [49]. The intensity, duration, frequency and extent of the occurrence are important contributors to the hazards of heat wave-related disasters [50]. Traditional temperature acquisition mainly involves meteorological stations, but meteorological data are acquired over a long period and limited in scope and cannot meet the requirements of large-scale spatial analysis. In contrast, the LST is an important factor in monitoring the dynamics of the resource environment, and several studies have indicated a linear relationship between the temperature and LST [51,52]. In conjunction with previous studies, an intensity indicator of the LST was selected to assess the hazard level of heat waves.

(2) Exposure

Exposure refers to “the presence of people, livelihoods, species or ecosystems, environmental functions, services, and resources, infrastructure, or economic, social, or cultural assets in places and settings that could be adversely affected” [49]. In this paper, risk assessment focused on the impact of risk on people, and therefore, only the population was selected as an exposure evaluation indicator. The more densely populated an area, the more severely it is likely to be affected by heat waves.

(3) Vulnerability

Vulnerability refers to “the propensity or predisposition to be adversely affected [and] encompasses a variety of concepts and elements, including sensitivity or susceptibility to harm and lack of capacity to cope and adapt” [49]. Infants and elderly individuals are considered heat-sensitive individuals due to their relatively low physiological capacity, resistance and immunity to disease and heat [5,53]. The economic level reflects the regional resilience and coordination during emergencies [28]. Previous studies have demonstrated that NTL data can effectively reflect the level of economic development [54]. Vegetation and water coverage plays an important role in regional cooling, and the level of vegetation and water coverage is closely related to the health risk of heat waves [55]. Numerous studies have indicated a significant positive correlation between the level of built-up land coverage and population heat-related morbidity and mortality [56]. Medical resources and their accessibility also constrain the average ability of people to resist and adapt to high temperatures from the top down. Therefore, based on the characteristics of heat waves and considering the availability of data, eight composite indicators were selected for vulnerability analysis: population of elderly individuals (>65 years), population of young individuals (<5 years), economic level, distance to a hospital, distance to a road, vegetation coverage, water coverage and impervious surfaces.

#### 2.3.2. Construction of HWR Assessment Model

(1) Data grading

To analyze the spatiotemporal variability in hazard, exposure, vulnerability and risk levels over time, the indicators must be graded according to a uniform standard (Table A1). This is accomplished by using the reclassification tool in ArcGIS to grade each indicator and assign a value from 1–5. The impervious surface data were binarized, and a value of 1 was assigned to impervious surfaces, while a value of 0 was assigned to pervious surfaces.

(2) Data normalization

To eliminate any differences in the magnitude, scale and attribute between the various indicators, the raw data are normalized via the polarization method before each index is calculated. The equations are expressed as follows (Equations (1) and (2)):

Positive indicators:

(1) $$R_{i}=\frac{x_{i}-x_{min}}{x_{max}-x_{min}}$$

Negative indicators:

(2) $$R_{i}=\frac{x_{max}-x_{i}}{x_{max}-x_{min}}$$

where $R_{i}$ denotes the normalized value of the *i*-th indicator (i=1,2,3.....,n), $x_{i}$ is the actual value of each indicator, and $x_{max}$ and $x_{min}$ are the maximum and minimum values, respectively, of each indicator.

(3) Calculation of each factor index

The hazard index (HI), exposure index (EI) and vulnerability index (VI) are calculated as follows (Equations (3)–(5)):

(3) $$HI=\frac{1}{n}\sum_{i=1}^{n}R_{i}$$

where $R_{i}$ denotes the normalized value of the *i*-th indicator in the hazard layer (i=1,2,3.....,n).

(4) $$EI=\frac{1}{n}\sum_{i=1}^{n}R_{i}$$

where $R_{i}$ denotes the normalized value of the *i*-th indicator in the exposure layer (i=1,2,3.....,n).

(5) $$VI=\frac{1}{n}\sum_{i=1}^{n}R_{i}$$

where $R_{i}$ denotes the normalized value of the *i*-th indicator in the vulnerability layer (i=1,2,3.....,n).

The risk index (RI) is calculated based on the above three risk components by using a weighted arithmetic approach, as expressed in Equation (6):

(6) $$RI_{i}=W_{H_{i}}\times HI+W_{E_{i}}\times EI+W_{V_{i}}\times VI$$

where $W_{H_{i}}$, $W_{E_{i}}$ and $W_{V_{i}}$ denote the weight values corresponding to the hazard, exposure and vulnerability, respectively, obtained from the *i*-th expert questionnaire.

By using 12 sets of relative weights derived from questionnaire surveys of 12 experts (Table A2), 12 risk assessment results were derived in the whole process [33]. The average value of the 12 risk assessment results was calculated to obtain the final heat wave risk index (HWRI).

(7) $$HWRI=\frac{1}{12}\sum_{i=1}^{n}RI_{i}$$

In the above equation, i=1,2,3.....,n, and n=12.

Finally, the corresponding layers for 2014 and 2019 were graded considering each index layer based on the thresholds determined in 2005 according to the natural interval method [41,57]. Each index layer was classified into five levels (very low, low, medium, high and very high), and the grading thresholds for each index layer are listed in Table A3.

## 3. Results

### 3.1. Analysis of the Spatiotemporal Variability in the Heat Wave Risk

#### 3.1.1. Hazard

**Figure 2:**
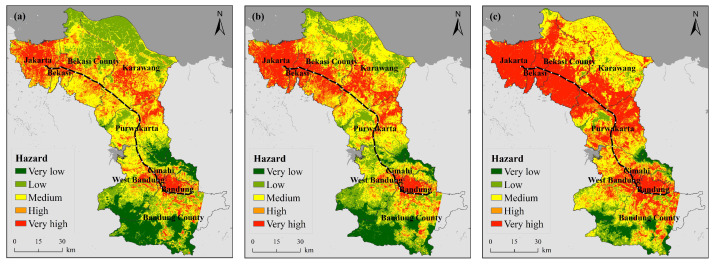
Spatiotemporal variation in the heat wave hazard ((**a**) 2005, (**b**) 2014, (**c**) 2019).

Figure 2 shows the spatial distribution of the regional heat wave hazards along the JBHSR from 2005 to 2019. In terms of the overall distribution of hazards, the areas with higher hazard levels were mainly located in Jakarta, Bekasi, Bandung and Cimahi. In terms of the overall trend, the hazard level indicated a significant increasing trend from 2005 to 2019.

Combined with Table A4, it is found that the proportion of very high-level hazards changed the most, increasing from 13.66% in 2005 to 39.48% in 2019, which is an increase of 25.82% over the past 15 years. The remaining four grades of hazards all indicated varying degrees of decreasing trends.

In conjunction with Figure 2, the observed variation was largely reflected spatially: (1) In the four cities of Jakarta, Bekasi, Bandung and Cimahi, the LST in most areas roughly remained above 35 °C in 2005, and at the center of these cities, the LST reached above 38 °C. By 2014, the range of the temperature distribution expanded outwards. By 2019, the LST in almost all areas of the four cities reached 38 °C or higher. The maximum temperature also increased from 44 °C in 2005 to 45 °C in 2014 and reached as high as 47 °C by 2019. This change mainly occurred because Jakarta is the economic, political and cultural center of Indonesia, and Bandung is the capital of West Java, while Bekasi and Cimahi, as two major cities in close proximity to both the capital and provincial capital, experienced a notable increase in population, with increasingly dense buildings and more vehicles over the past 15 years, resulting in increasingly pronounced urban heat island effects. (2) The changes in Bekasi, Karawang and Puwakarta counties were also noteworthy. Due to their proximity to the Java Sea and the influence of the sea and monsoons, northern Bekasi and northern Karawang regencies attained a lower LST than that of inland areas. The region exhibited a low risk level in 2005, a medium risk level in certain areas by 2014, and a medium risk level in almost all areas by 2019, with scattered areas even indicating a very high risk level. Southern Bekasi and southern Karawang counties, in addition to northern Puwakarta County, revealed large contiguous areas with the LST reaching or even exceeding 38 °C in 2019. Combined with the land cover, it is clear that these areas are predominantly urban areas which have also experienced high urbanization rates and the consequent accelerated LST increases during the fifteen-year period of interest.

#### 3.1.2. Exposure

**Figure 3:**
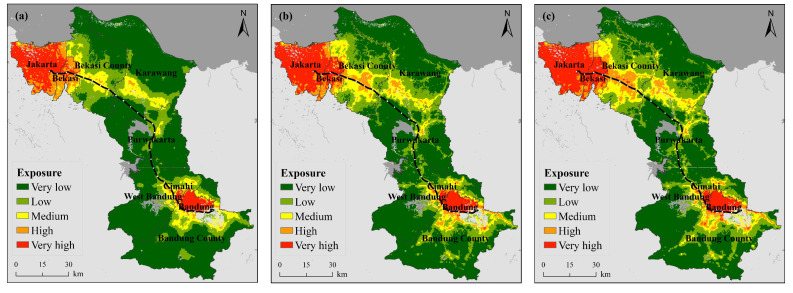
Spatiotemporal variation in the heat wave exposure ((**a**) 2005, (**b**) 2014, (**c**) 2019).

Figure 3 shows the spatial distribution of heat wave exposure along the JBHSR from 2005–2019. Humans are the main exponents of heat wave disasters, and the more densely populated a given area, the more severe the impact and the higher the exposure level will be. In terms of the overall exposure distribution, the areas with a higher exposure are mainly located in Jakarta, Bekasi, Bandung and Cimahi. In terms of the overall trend, the exposure levels indicate an increasing trend from 2005 to 2019.

Combined with Table A5, the proportion of the very low exposure level changed the most, decreasing from 61.62% in 2005 to 48.99% in 2019, a total decrease of 12.63%. The remaining four exposure grades maintained a slow increasing trend, with the change in each grade reaching 5.85% (low), 1.66% (medium), 1.83% (high) and 3.30% (very high).

Spatially, this change is mainly evident in central Bekasi County, central Karawang County and northern Bandung County. The central areas of these three counties host numerous towns, and these areas have gradually shifted over the past 15 years from areas with low exposure levels to areas with medium and high exposure levels as the population has grown and cities have expanded. In Bekasi and Bandung counties, there are even scattered areas with a very high exposure level. Exposure has also increased in Jakarta, Bekasi, Cimahi and Bandung. Combined with the demographic statistics of Indonesia, this change can be explained by the fact that the Indonesian population has grown over the years, and the population of Indonesia reached approximately 263 million people by 2017. Moreover, 60% of the population is concentrated on the island of Java, which is host to a population of 145 million people (2015) and a density of 1121 people per square kilometer, making the island the most populous Indonesian island and one of the most densely populated islands globally. The increasing number of people and the population density in the study area has resulted in a trend of increasing exposure to heat waves across the region.

#### 3.1.3. Vulnerability

**Figure 4:**
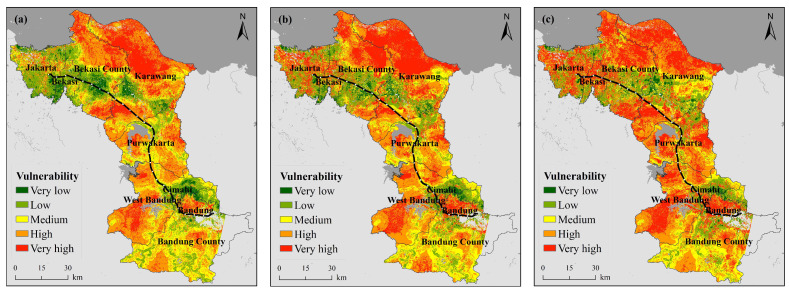
Spatiotemporal variation in the heat wave vulnerability ((**a**) 2005, (**b**) 2014, (**c**) 2019).

Figure 4 shows the spatial distribution of the heat wave vulnerability along the JBHSR from 2005–2019. In terms of the overall vulnerability distribution, most areas exhibit high vulnerability levels. In terms of overall trends, the vulnerability level indicates a notable increasing trend from 2005 to 2019.

In conjunction with Table A6, the proportion of the very high vulnerability level changed the most, increasing from 20.31% in 2005 to 35.13% in 2019, an increase of 14.82% over the past 15 years. The remaining four vulnerability grades all demonstrated varying degrees of decreasing trends.

The reasons for the change in vulnerability include, on the one hand, the overall lack of health care resources, and on the other hand, the economic level, although an overall growth trend is maintained. Notable vulnerability variation is concentrated in large cities such as Jakarta and Bandung, with a much lower economic growth level in suburbs far from cities than the per capital level. Road facilities are quite poor, with only one highway between the two major cities of Jakarta and Bandung and only one light rail line in Jakarta, which opened in 2019, resulting in unbearable traffic jams and preventing timely access to medical treatment in the event of heat waves. Continued city expansion has encroached on previously cultivated grassland and woodland areas, thereby reducing the vegetation coverage and increasing the urban building coverage, further exacerbating the urban heat island effect. Of particular interest is Jakarta, where the overall vulnerability level was low in 2005, but this city has changed the most notably over the last 15 years, with most of Jakarta attaining a relatively high vulnerability level in 2019. The city is densely populated and contains relatively abundant medical resources. While the medical resources in large cities have improved the human capacity to respond to disasters to a certain extent, this has been accompanied by the reality of an increasingly aging population and rising birth rates, which have exerted much pressure on these cities. In summary, from 2005–2019, despite a slight increase in health care resources and an increase in economic level, the overall vulnerability still increased due to multiple factors. Therefore, there is an urgent need to identify the contributing factors as a means of reducing the impact of heat waves on humans.

#### 3.1.4. Risk

**Figure 5:**
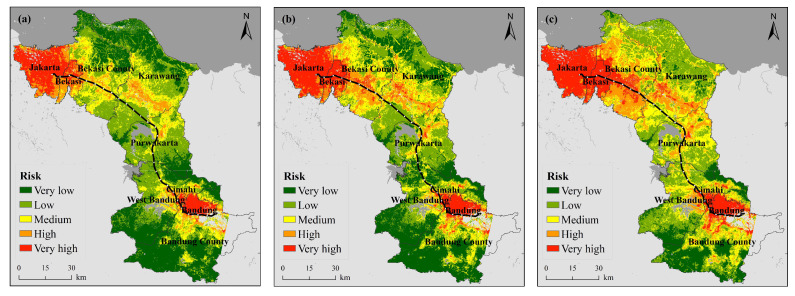
Spatiotemporal variation in the heat wave risk ((**a**) 2005, (**b**) 2014, (**c**) 2019).

Figure 5 shows the spatial distribution of the HWR along the JBHSR from 2005–2019. Overall, there occurs significant spatial variation in the HWR. Jakarta achieves the highest risk level. In terms of the overall dynamics, the risk level notably increases from 2005 to 2019.

Based on Table A7, all four risk grades notably varied except the low risk level. Among these levels, the very low risk level changed the most, decreasing from 31.16% in 2005 to 11.47% in 2019, a decrease of 19.69% over 15 years.

Combined with Figure 5, the following is observed in space: (1) the above changes are mostly reflected in the four larger cities of Jakarta, Bekasi, Bandung and Cimahi. In 2005, although most areas occurred at a very high risk level, a high risk level was observed in suburbs farther away from the city center, but by 2019, almost all areas of the four cities had reached a very high risk level. The main reasons for this change include the increasing number of buildings, the development of large amounts of arable grassland for building purposes, the increase in vehicles within the city and the emission of gases such as carbon dioxide, all of which cause higher heat accumulation in the city, a more severe urban heat island phenomenon and a rapid increase in the LST. City development has also attracted more people, thus increasing the exposure to heat waves once again. The level of medical care and various infrastructures within cities yield a major advantage over rural areas, resulting in a large aging population, with a relatively high birth rate and therefore an increasing vulnerability. The combination of these effects has resulted in an overall increasing risk level in the region. (2) Areas of concern also include the interior of Karawang, Bekasi and Puwakarta counties, all of which contained scattered small areas with a high risk level in 2005. By 2014, high-level risk areas had expanded outwards, resulting in contiguous areas and scattered areas with a very high risk level. By 2019, this expansion trend continued, and contiguous areas with a very high risk level emerged. Combined with the land cover, the scattered small high-grade areas in 2005 encompassed urban areas, covered by a large number of buildings and surrounded mostly by cultivated grasslands, while in the northern part of Karawang and Bekasi counties, farmers took advantage of the unique geographical location to create many fish ponds and salt pans. Economic development has led not only to urban expansion in large cities but also to expansion and development of urban areas, conversion of large amounts of arable forestland into construction land, reduction in vegetation coverage and population growth in the area, with a number of factors leading to an increasing HWR.

### 3.2. Comparative Analysis of the Heat Wave Risk Results

By comparing the results with the 2015 global standard heat wave index distribution of Raei [50] and the Belt and Road regional HWR assessment of Yin [21], it is concluded that the regional HWR at the kilometer scale agrees well with that determined in previous large-scale studies in terms of the overall dynamics. The risk is higher in the northwestern part of the study area and the highest in the Jakarta region.

**Figure 6:**
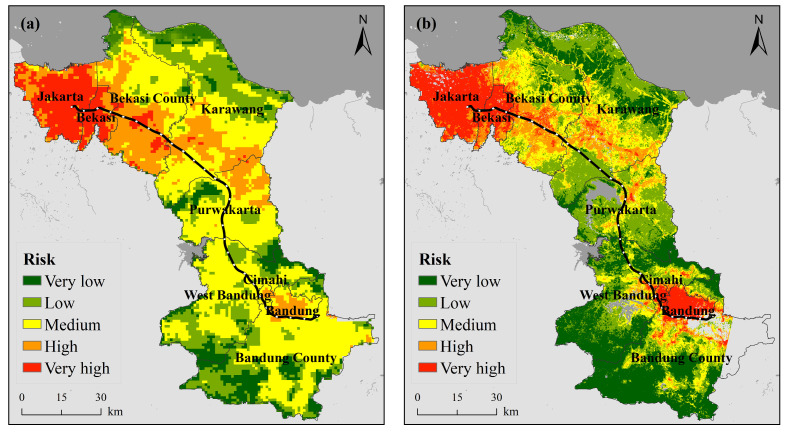
Heat wave risk distribution map ((**a**) 2015 km scale, (**b**) 2014 hundred meter scale).

**Figure A2:**
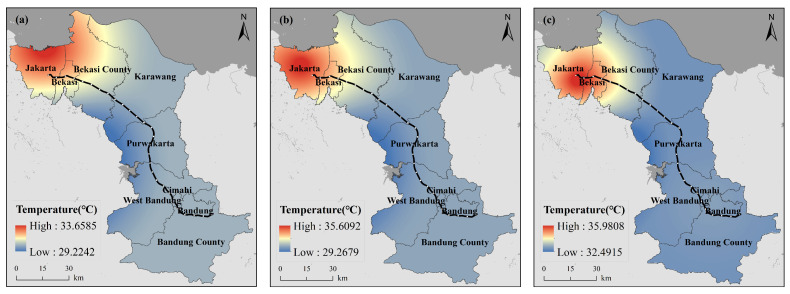
Map of the daily maximum temperature ((**a**) 4 September 2005, (**b**) 13 September 2005, (**c**) 11 September 2005).

**Figure A3:**
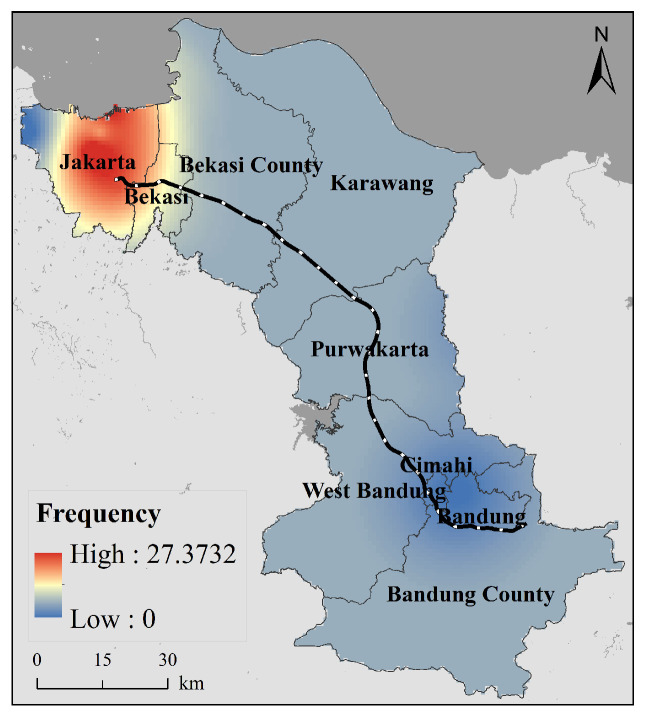
Map of frequency of historical heat waves.

Despite the differences in assessment methods, data, and indicator selection and grading standards, the results of this assessment inevitably differ from previous research results. However, in terms of the overall risk trend, the obtained HWR assessment results at the 100-m scale agree well with those at the kilometer scale (Figure 6). To further validate the results of this paper, Figure A2 and Figure A3 show interpolated daily maximum temperatures and historical heat wave frequency values determined based on meteorological station data, which shows that the risk level in Jakarta in the northeast is notably higher than that in the other regions. In summary, the findings of this paper are credible.

### 3.3. Analysis of the Heat Wave Risk Variation Based on the Subdistrict

**Figure 7:**
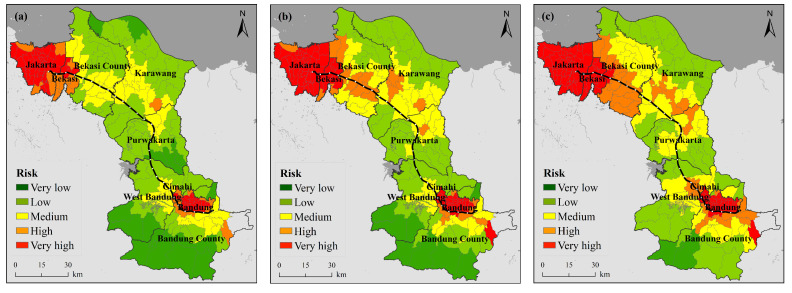
Map of the subarea-scale heat wave risk level ((**a**) 2005, (**b**) 2014, (**c**) 2019).

ArcGIS was applied to spatially link HWR raster data with subdistrict vector data to obtain a map of the HWR levels at the subdistrict scale (Figure 7).

The risk level zoning results indicate that there are 205 administrative units at the subdistrict scale (Table A8 and Table A9). Sixty-nine zoning units occurred at a very high risk level in 2005, increasing to 84 in 2014 and 91 in 2019, while the number of zoning units in very high-risk areas accounted for 44.39% of all zoning units by 2019. The very high-risk areas were concentrated in Jakarta, Bekasi, Cimahi and Bandung, and the high-risk areas exhibited an expansion trend toward surrounding areas over the past 15 years. By 2019, all three cities of Jakarta, Bekasi and Cimahi had attained a very high risk level. These areas are characterized by dense populations, high concentrations of urban buildings, more frequent summer heat processes and heat wave events, and a higher human health risk associated with hot weather conditions. Moreover, although medical resources are relatively abundant in these areas, the total amount remains insufficient to alleviate the summer heat risk.

### 3.4. Heat Wave Risk Analysis Based on High-Speed Rail Stations

**Figure 8:**
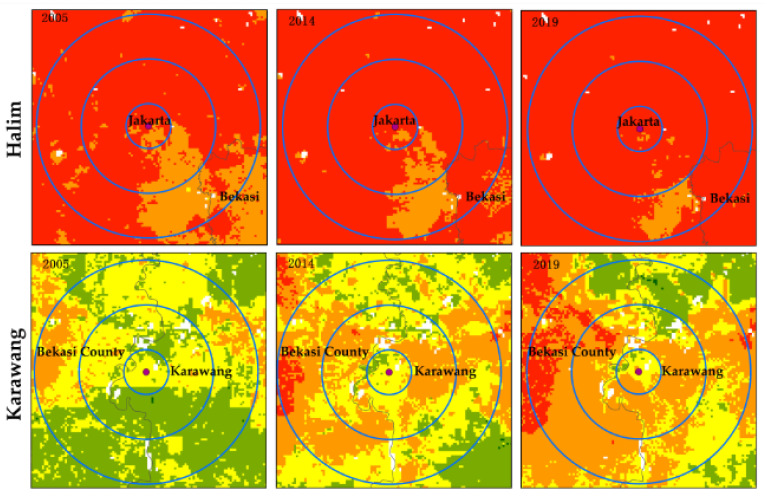

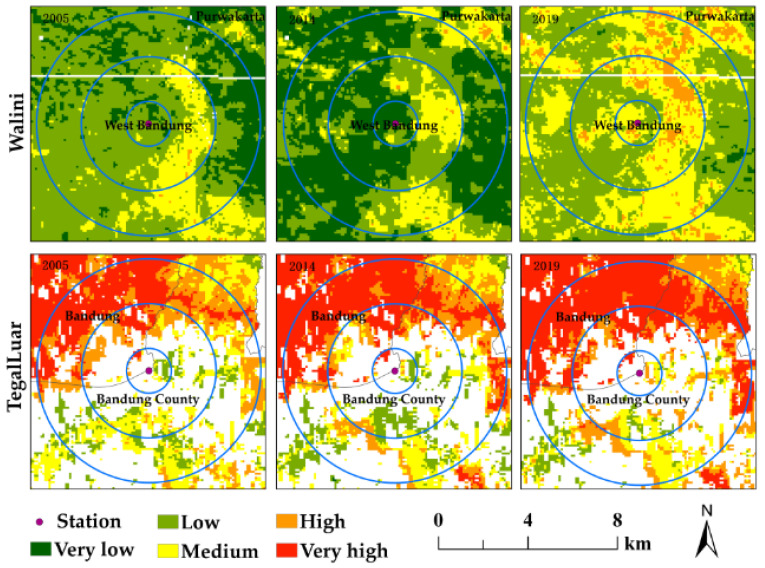
Spatiotemporal variation in the heat wave risk around each Jakarta-Bandung high-speed railway station from 2005 to 2019.

The JBHSR line currently encompasses four high-speed railway stations, namely, Harlem, Karawang, Walini and TegalTuar. Analysis of the change trends of the HWR near these stations can provide scientific recommendations for urban structure planning and adaptation measures along the JBHSR. We adopt ArcGIS to generate multiring buffer zones centered on the above stations and overlay risk layers to determine the risk change trend in 1-, 3- and 5-km areas around each station. According to Figure 8, overall, the risk around each station demonstrates an increasing trend from 2005–2019. The risk level around each station reveals the following sequence: Halim > TegalTuar > Karawang > Walini.

### 3.5. Identification and Analysis of the Driving Factors of the Heat Wave Risk

**Figure 9:**
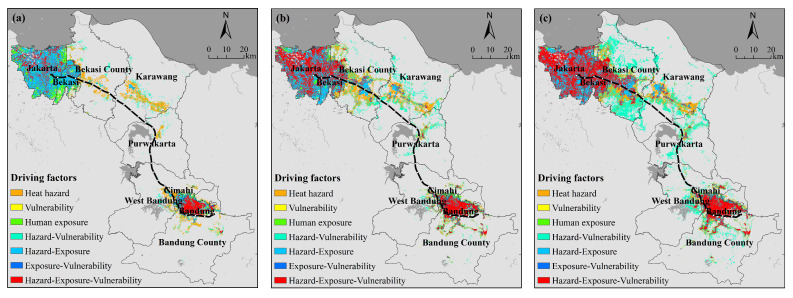
Leading factors of the heat wave risk ((**a**) 2005, (**b**) 2014, (**c**) 2019).

In addition to the identification of high-risk areas, it is important that decision makers recognize the risk factors that play a dominant role in shaping these high-risk areas. Here, pixels with high and very high risk levels are considered potential high-risk areas. The hazard, exposure and vulnerability indices were also reclassified into two classes in the same manner. The main drivers leading to potential high-risk areas along the JBHSR were thus identified (Figure 9). For example, the H-V (hazard-vulnerability) label indicates that both the hazard and vulnerability levels are high in the corresponding area, while the exposure level is low. Therefore, the aspects of hazard and vulnerability are defined as drivers of high risk.

As indicated in Table A10, risk areas driven by a single factor exhibited a decreasing trend over the last 15 years. The majority of the high-risk areas were influenced by high-temperature hazards and revealed an increasing trend over the past 15 years. There are three types of high-risk areas that greatly changed and accounted for a high proportion of the risk areas: areas influenced by the synergistic hazard-exposure-vulnerability effect, areas influenced by the synergistic hazard-exposure effect, largely located in Jakarta, Bandung, Bekasi and Cimahi, and areas influenced by the synergistic hazard-vulnerability effect, mainly located at the centers of certain districts.

## 4. Discussion

Previous studies have shown that there is an urgent need for more spatial specificity in HWR assessment under a geospatial framework [5,58,59]. In this study, the remote sensing-based HWR assessment method can demonstrate the distribution of risk at the pixel level. Moreover, this paper conducts research based on the 100-m scale, which effectively improves the spatial granularity of the data compared to existing studies (in which provinces, cities, or counties were the smallest research units), and the maps are more informative and intuitive, which is more helpful for communicating and understanding specific human risks. To the best of our knowledge, this is the first HWR map along the JBHSR, and the first spatial and temporal map of HWR at the 100-m scale in the world based on the region along the high-speed railway and taking into account the distribution of the natural and socio-economic environments. With limited cost, time, and labor, this study is particularly valuable in guiding local decision makers to proactively develop adaptation strategies for mitigation interventions and climate impacts [60]. The method can also provide new ideas for HWR assessment in countries along the Belt and Road.

In the absence of spatially explicit population distributions, previous studies have opted to use a proxy index, called the elevation-adjusted human settlement index, which can be produced by using a set of remotely sensed data, including a nighttime lights dataset, a vegetation index, and a digital elevation model [5,14,41]. Fortunately, the downscaling of population data has improved in recent years. Global gridded population data are now available at various spatial resolutions and time points. For instance, WorldPop provides gridded population data products with a spatial resolution of 100 m for the corresponding year [44]. Estoque et al. have demonstrated that this dataset has a significant positive correlation with census data ($R^{2}$ = 0.9926) [33]. This paper takes full advantage of this dataset to obtain finer spatialized population data, which not only improves the accuracy of the overall risk assessment but can also effectively overcome the difficulty of obtaining detailed socioeconomic statistics for many countries, and can provide a new way to obtain data for HWR assessment in the countries along the Belt and Road.

Most previous studies have focused on quantifying heat vulnerability and heat health risks in urban environments. This study takes a different approach by selecting the strategically important area along the JBHSR, which has a complex landscape and passes through towns with different levels of development and different sociodemographic population characteristics. The 2005 vulnerability map of the region along the JBHSR (Figure 5) is consistent with studies by Sheridan and Dolney [61], Wu et al. [62], Henderson et al. [63] and Hu et al. [5] that show that rural populations are more vulnerable to extreme heat than urban populations. Urban areas are usually better able to adapt to extreme heat than rural areas, probably because they have higher socioeconomic status and better medical resources [62]. In addition, a population cross-sectional survey in Guangdong Province, South China, showed that rural populations have very low perceptions of HWR and rarely take adaptive measures during heat waves [64]. The vulnerability maps for 2014 and 2019 (Figure 5) for this study area show high vulnerability not only in rural areas but also in urban areas, which is consistent with the study by Aubrecht and Ozceylan [65], which showed high vulnerability in the U.S. Capital Region. Vulnerability shows an overall trend of increasing over time. The explanations of this trend may be diverse: first, as cities grow, green spaces decrease and impervious surfaces increase, making urban heat islands more severe; second, due to data limitations, road and hospital data, the constant 2014 data (OpenStreetMap) was used, which has an impact on the assessment of dynamic changes in vulnerability.

For indicator weights, this paper uses a combination of equal weights and relative weights. Although the literatures present different contributions of environmental, demographic, and socioeconomic indicators, there is not yet an accepted standard weight for each indicator [66,67]. Therefore, most previous studies have assumed equal importance and set equal weights. In this paper, equal weights are used within specific risk components. In addition, in calculating the risk, this paper uses a relative weight based on expert judgment and is obtained by Estoque et al. through a questionnaire filled out by experts in several fields, with a certain degree of objectivity and reliability [33].

However, there remain certain aspects that should be improved in this paper. (1) In terms of hazards, this paper employs the LST, which only considers the effect of temperature on humans, but the effect of heat waves on humans is also influenced by the relative humidity and wind speed [28]. Furthermore, the hazard indicators selected in this paper only include the heat wave intensity, while the heat wave frequency and duration are also crucial in the heat wave process. In future studies, we will consider apparent temperature data to characterize heat wave hazards [68], and we will add frequency and duration indicators. (2) The lack of detailed data also imposes some limitations on the study presented in this paper, for example, heat sensitivity data (e.g., people’s physical health status) or adaptive capacity data (e.g., air conditioning ownership, education or literacy rates, etc.). However, all of these indicators mentioned, including those considered relevant but not mentioned, need to be explored and will be considered for inclusion in future updates of this study once city-level or more detailed-level data are available. (3) Heat waves can be fatal in severe cases, so the number of heat-related deaths or the incidence of heat-related mortality best validates the HWR [5,21], but these data are currently difficult to obtain. The extraction of heat-related mortality data from all-cause mortality data and mortality data related to various diseases will be a key focus of future HWR studies. (4) Although Landsat data provide a high spatial resolution and can be applied in fine-scale studies, due to their low temporal resolution, the two images obtained in this paper pertaining to the same period (the same day) do not completely cover the entire study area, resulting in a missing lower right corner of the research results in this study. Therefore, data acquisition at a high spatial resolution and high coverage will also become a focus of further exploration, and future research will attempt to solve this problem based on MODIS data, combined with downscaling algorithms. (5) The importance of each assessment factor (population of elderly individuals, population of young individuals, economic level, hospital/road distribution, vegetation/water coverage, impervious surface) was not differentiated when conducting the vulnerability assessment. In fact, each factor contributes differently to the heat wave risk [21,41]. However, there is currently no uniformity in the determination of the weights of each indicator in heat wave risk evaluation. How to determine more objective and reasonable indicator weights when each influencing factor has a different degree of influence on the overall risk evaluation will also be the focus of future research on risk assessment.

Furthermore, (1) The resolution of NTL data used in this paper is 1 km. Chen published the latest NTL data, which is a long time series with 500 m resolution [69]. In future research, the 500 m resolution NTL data will be used to replace the 1 km NTL data, which will further improve the evaluation accuracy. (2) The spatial advantage of surface temperature data is more prominent; however, its temporal accuracy is lower and it is more seriously affected by clouds. The time series of meteorological station data is more complete, but its spatial accuracy is limited and cannot reflect the spatial variability of temperature in a region [23,48]. In the future, we will try to combine RF (Random Forest) and LSTM (Long Short-term Memory) algorithms to build a fine-scale temperature dataset with a complete time series. Using this dataset to calculate heat waves will also increase the accuracy of the data to a certain extent, thereby improving the accuracy of the risk assessment results. (3) The study of spatiotemporal changes in the HWR over long time series is of great practical significance in revealing the evolution of risk hotspots, HWR development trends and disaster response and prevention patterns. In future studies, the time series will be further increased to include years before 2005 (2000, 1995, 1990, etc.) to explore the spatiotemporal variation in the HWR over a longer time series. Given a longer time series, the impact of additional sensitivity indicators (GDP, population, etc.) on the HWR can be further clarified. Moreover, we will perform a long-term follow-up assessment of the HWR in the region after JBHSR completion in the future. This study can provide a good reference case for research on the changes in HWR caused by regional development along high-speed railways in more countries, which is highly important. (4) Combined with the changes in population size and GDP per capita over the last 50 years in Indonesia, it is clear that the influencing factors have notably changed over the last 50 years and that with rapid socioeconomic development, the rate of increase in the population size and GDP will further increase and heat waves will become more severe. Therefore, in future HWR studies, the analysis frequency should be increased, and a time interval of three years, two years, or even one year should be chosen in the examination of spatiotemporal change patterns. (5) Combined with previous studies, it is predicted that the excess mortality of the population of elderly individuals due to heat waves will notably increase against the background of global warming [14,29]. However, with social progress and the increasing availability of healthcare resources, population aging is becoming a common phenomenon worldwide. Therefore, in future studies on HWR assessment, there is a need to not only analyze the overall risk of elderly individuals as a highly sensitive population group but also to further explore the relationship between heat and excess mortality among elderly individuals and to formulate scientific recommendations to mitigate the impact of heat waves on this demographic group.

## 5. Conclusions

Assessments of extreme heat vulnerability and risk are mostly studied for a single heat wave or on a one-year time scale, but HWR assessment on long time scales is rarely carried out. Within the general framework of the main determinants of risk (hazard, exposure, and vulnerability), we conducted a comprehensive, spatially explicit, and long time series analysis to assess the HWR and the spatiotemporal variability of the risk along the JBHSR. Multi-sensor remote sensing data, demographic and socioeconomic data, and geographic information system (GIS) technology were used to calculate the HWRI and to develop a raster map of the spatial distribution of HWR at the 100-m scale for the last 15 years (2005, 2014, and 2019). Heat wave risk is spatially highly variable. The higher risk areas are concentrated in Jakarta, Bandung, Bekasi and Cimahi, which mainly reflect the urban heat island effect and population exposure. Heat wave risk shows a significant upward trend from 2005–2019. Second, we qualitatively validated the regional HWR distribution by combining the results of previous research on a large scale and risk assessment at the kilometer scale, and the results showed good agreement with previous studies in terms of overall distribution trends. Finally, we explored the driving factors for the high-risk region, and the results showed that the study area was most severely affected by the heat hazard.

Our findings show that heat hazard is the most significant contributor to higher risk areas within the experimental study area, especially in large cities such as Jakarta (Figure 9). It was also found that the areas of high risk caused by a single factor are decreasing, and the areas of high risk caused by the synergistic effect of risk, exposure, and vulnerability are increasing (Table A10). In light of these findings, it is recommended that, on the one hand, measures to reduce or mitigate the intensity of heat hazards be considered, and in this regard, various measures related to urban development (e.g., the use of highly resilient materials in building design, the use of cool-toned materials on roofs and streets, and the implementation of urban greening strategies) are current research priorities in this field [70,71]. On the other hand, optimizing the structure of town construction, increasing medical resources, and increasing infrastructure such as summer facilities can be considered.

## Data Availability

The raw data supporting the conclusions of this article are available on request from the authors.

## Abbreviations

The following abbreviations are used in this manuscript:

HWR	Heat Wave Risk
JBHSR	Jakarta-Bandung high-speed railway

## Appendix B

**Table A1:** Grading standards for each indicator.

Indicators	1	2	3	4	5
LST (℃)	<5	25∼30	30∼35	35∼38	>38
Population count	<10	10∼25	25∼50	50∼100	>100
MNDWI	−1∼0	0∼0.2	0.2∼0.4	0.4∼0.6	0.6–1
NDVI	−1∼0.2	0.2∼0.4	0.4∼0.6	0.6∼0.8	0.8–1
>65 years	<2	2∼4	4∼6	6∼8	>8
<5 years	<4	4∼8	8∼12	12∼16	>16
Economic level (DN value)	0∼13	13∼24	24∼39	39∼53	53∼63
Distance to a road (m)	0∼500	500∼1000	1000∼3000	3000∼5000	>5000
Distance to a hospital (m)	0∼1000	1000∼3000	3000∼5000	5000∼8000	>8000

**Table A2:** Relative weight values [33].

Components	AHP Weights											
	1	2	3	4	5	6	7	8	9	10	11	12
Hazard	0.10	0.65	0.16	0.08	0.07	0.06	0.10	0.13	0.33	0.13	0.09	0.74
Exposure	0.64	0.07	0.30	0.66	0.68	0.60	0.64	0.60	0.33	0.46	0.32	0.08
Vulnerability	0.26	0.28	0.54	0.26	0.25	0.35	0.26	0.28	0.33	0.42	0.59	0.18

**Table A3:** Grading thresholds for each factor in 2005.

Grades	Very Low	Low	Medium	High	Very High
Hazard	0–0.2	0.2–0.4	0.4–0.6	0.6–0.8	0.8–1
Exposure	0–0.2	0.2–0.4	0.4–0.6	0.6–0.8	0.8–1
Vulnerability	0–0.3103	0.3103–0.4055	0.4055–0.4673	0.4673–0.5290	0.5290–1
Risk	0–0.2336	0.2336–0.3482	0.3482–0.4923	0.4923–0.6790	0.6790–1

**Table A4:** Changes in the percentage of the different hazard levels from 2005–2019 (%).

Year	Very Low	Low	Medium	High	Very High
2005	12.21%	23.68%	33.35%	17.09%	13.66%
2014	9.67%	21.51%	33.11%	15.97%	19.73%
2019	4.43%	8.82%	30.77%	16.49%	39.48%

**Table A5:** Changes in the percentage of the different exposure levels from 2005–2019 (%).

Year	Very Low	Low	Medium	High	Very High
2005	61.62%	16.97%	8.34%	5.00%	8.06%
2014	52.47%	21.90%	9.55%	5.84%	10.24%
2019	48.99%	22.82%	10.00%	6.82%	11.37%

**Table A6:** Changes in the percentage of the different vulnerability levels from 2005–2019 (%).

Year	Very Low	Low	Medium	High	Very High
2005	4.10%	17.85%	25.12%	32.62%	20.31%
2014	3.49%	15.34%	23.86%	29.23%	28.07%
2019	2.59%	12.97%	21.11%	28.20%	35.13%

**Table A7:** Changes in the percentage of the different risk levels from 2005–2019 (%).

Year	Very Low	Low	Medium	High	Very High
2005	31.16%	32.38%	18.12%	9.28%	9.07%
2014	26.52%	29.25%	19.63%	11.79%	12.82%
2019	11.47%	30.95%	25.14%	16.39%	16.04%

**Table A8:** Number of subdistricts at each heat wave risk level.

Year	Very Low	Low	Medium	High	Very High
2005	16	58	37	25	69
2014	8	50	39	24	84
2019	2	41	41	30	91

**Table A9:** Number of subdistricts at each heat wave risk level based on city/county.

City/County	Year	Very Low	Low	Medium	High	Very High
Jakarta	2005	0	0	0	5	40
	2014	0	0	0	1	44
	2019	0	0	0	0	45
Bandung	2005	0	1	0	5	24
	2014	0	1	0	4	25
	2019	0	1	0	2	27
Bekasi	2005	0	0	0	8	4
	2014	0	0	0	2	10
	2019	0	0	0	0	12
Cimahi	2005	0	0	0	2	1
	2014	0	0	0	0	3
	2019	0	0	0	0	3
Bekasi country	2005	0	13	10	0	0
	2014	0	6	10	7	0
	2019	0	1	9	12	1
Karawang	2005	2	17	10	1	0
	2014	0	16	10	4	0
	2019	0	17	9	4	0
Purwakarta	2005	3	11	4	0	0
	2014	0	10	7	1	0
	2019	0	8	8	2	0
Bandung country	2005	8	7	9	4	0
	2014	5	8	8	5	2
	2019	2	6	9	8	3
West Bandung	2005	3	9	4	0	0
	2014	3	9	4	0	0
	2019	0	8	6	2	0

**Table A10:** Share of each influencing factor from 2005–2019.

	Hazard	Exposure	Vulnerability	H-E	H-V	E-V	H-E-V
2005	19.16%	10.96%	0.40%	41.39%	7.86%	0.97%	19.26%
2014	16.97%	4.01%	0.64%	28.36%	16.18%	0.81%	33.02%
2019	13.10%	1.14%	0.17%	20.65%	29.85%	0.19%	34.91%
